# Prevalence of syphilis in transgender women and travestis in Brazil: results from a national cross-sectional study

**DOI:** 10.1590/1980-549720240003.supl.1

**Published:** 2024-08-19

**Authors:** Aline Borges Moreira da Rocha, Sandro Sperandei, Adele Benzaken, Rita Bacuri, Katia Cristina Bassichetto, Elaine Lopes de Oliveira, Edilene Peres Real da Silveira, Maria Inês Costa Dourado, Maria Amélia de Sousa Mascena Veras

**Affiliations:** I Santa Casa de São Paulo, School of Medical Sciences – São Paulo (SP), Brazil.; II Western Sydney University, Translational Health Research Institute – Sydney (NSW), Australia.; III Fiocruz Amazônia, Instituto Leônidas e Maria Deane – Manaus (AM), Brazil.; IV Aids Healthcare Foundation – Los Angeles (CA), EUA.; V Instituto Adolfo Lutz – São Paulo (SP), Brazil.; VI Universidade Federal da Bahia, Institute of Public Health – Salvador (BA), Brazil.

**Keywords:** Syphilis, Transgender women, Prevention, Point-of-care, Testing and treating

## Abstract

**Objective:**

The study aimed to estimate the prevalence of acquired syphilis and associated factors in a national survey.

**Methods:**

TransOdara was a cross-sectional study comprising transgender women and *travestis* (TGW) in five major cities in Brazil during December of 2019 and July of 2021. The sample was recruited using the *respondent-driven sampling* (RDS) method. The outcome “active syphilis” was defined as a positive treponemal test and Venereal-Disease-Research-Laboratory (VDRL) title greater than∕ equal to ⅛. Sociodemographic variables were described. Bivariate and multiple logistic regression were performed, and odds ratios (OR) and 95% confidence intervals (95%CI) were estimated. All analyses were performed in R, 4.3.1.

**Results:**

A total of 1,317 TGW were recruited, with 1,291 being tested for syphilis, and 294 (22.8%) meeting the criteria for active syphilis. In bivariate analysis, black/mixed race (OR=1.41, 95%CI 1.01–1.97), basic level of education (OR=2.44, 95%CI 1.17–5.06), no name change in documents (OR=1.39, 95%CI 1.00–1.91) and sex work (past only OR= 2.22, 95%CI 1.47–3.32; partial OR=2.75, 95%CI 1.78–4.25; full time OR=3.62, 95%CI 2.36–5.53) were associated with active syphilis. In the multivariate analysis, sex work was the only associated factor, 2.07 (95%CI 1.37–3.13) past sex work, 2.59 (95%CI 1.66–4.05) part-time sex work and 3.16 (95%CI 2.04–4.92) sex work as the main source of income.

**Conclusion:**

The prevalence of active syphilis in this study was elevated compared with other countries in Latin America. Sex work was an important associated factor with active syphilis, highlighting the impact that this condition of vulnerability may have in the health of TGW, as members of a key, marginalized population.

## INTRODUCTION

Sexually transmitted infections (STIs) remain a common condition of health and affect different populations worldwide. Syphilis constitutes an important health issue to be addressed, considering reports of increasing rates, limited access to rapid tests (point of care tests — POCT) in some countries, and multiple phases of clinical manifestation, frequently of concern for patients, their partners and newborns^1^. In Brazil, recent data from the national surveillance system reported 1,115,529 cases of acquired syphilis between the years of 2011 and 2021, with notifications concentrated in young males and constantly rising detection rates: 9.3 cases per 100 thousand inhabitants in 2011 and 78.5 cases per 100 thousand inhabitants in 2021^2^. It is worth mentioning that Brazil´s surveillance system does not collect data on gender identity.

Transgender women and *travestis* (TGW) face a high burden of human immunodeficiency virus (HIV) and other STIs, including syphilis, a condition that often goes undiagnosed mainly due to the difficulty of accessing health services^3^. In Latin America, data on syphilis among the transgender population are scarce. One national survey found a prevalence of 47.4% in the Dominican Republic and a retrospective chart review study found 54.8% in Lima, Peru^4,5^. A few Brazilian studies have addressed the prevalence of syphilis among the TGW population, with data ranging from 33.3% in a PrEP cohort study among adolescents conducted in three Brazilian capitals between 2019 and 2021, and 50.0% in a cross-sectional study conducted with TGW in Central Brazil in 2014^6,7^.

Among the factors associated with a higher prevalence of syphilis among TGW, low socioeconomic status and sex work emerge as the most important ones. These conditions are closely related to the situation of social vulnerability to which a substantial part of this population is subjected, making access to health care services difficult, especially those related with sexual health^3,8^.

The present study estimates the prevalence of acquired syphilis and associated factors among TGW in Brazil.

## METHODS

TransOdara was a cross-sectional survey comprising TGW conducted in five major cities across all regions in Brazil: Campo Grande (MS), Manaus (AM), Porto Alegre (RS), Salvador (BA) and São Paulo (SP), between November 2019 and July 2021.

TGW were recruited using the respondent-driving sampling (RDS) method, an approach used to achieve hard-to-reach populations that relies on social networks to recruit members of the same population^9^. For this study, recruitment took place between December 2019 and July 2021, considering the possible variations in each site due to the enormous impact of the COVID-19 pandemic in all aspects of the health care services.

All the participants answered a structured questionnaire about sociodemographics, their experience of stigma and discrimination and previous knowledge about HIV and other STIs, including information about previous testing and treatment of each one of them. Rapid blood tests for HIV, syphilis and hepatitis A, B and C were offered, in addition to a real-time PCR exam (Abbott Real Time CT/NG Controls) for *Neisseria gonorrhoeae* and *Chlamydia trachomatis,* in the samples of self-collected anal e genital/urethral swabs, with confirmatory tests being performed in case of a positive result and offering treatment to confirmed cases. In the case of symptomatic patients for different STIs, after collecting specimens, a syndromic approach was carried out, based on the Ministry of Health’s guidelines, and the appropriate treatment was prescribed. For more methodological details of the study, see the methodological article about TransOdara published in this same supplement^10^.

Study variables: For the outcome ‘active syphilis’, blood samples were tested at the Adolfo Lutz Institute, a reference public health laboratory for the state of São Paulo. For syphilis diagnosis, a rapid (treponemal) point of care test (POCT) provided by the State Health program, and a non-treponemal test Venereal Disease Research Laboratory (VDRL) were used for confirmation. In case of inconsistent results (negative POCT and positive VDRL), a second treponemal test (fluorescent treponemal antibody absorption test — FTA-Abs) was performed to confirm the results. Participants who presented a positive treponemal test and VDRL titles greater than or equal to ⅛ were classified as having active syphilis for this analysis, and received treatment. Due to the difficulty of characterizing the clinical follow-up of participants with VDRL titles less than ⅛, these individuals also received treatment, in accordance with the Ministry of Health manual for treatment of STIs^11^.

Associated factors included in the analyses were age (up to 34 years vs. 35 or more), marital status (single, including separated, divorced or widowed, in a stable relationship but not married, and married), race/skin color (white, black or mixed, and other), highest educational attainment (basic, high school, and higher education), housing arrangement (owner, leasing, with friends or family, and other, including homeless, hotels, and institutions), monthly income (less than one minimum wage, one to two minimum wages, and more than two minimum wages — considering the minimum wage equivalent to 218.29 US dollars), history of sex work (never, past only, currently part-time source of income, and currently main source of income), history of violence, history of physical assault, history of sexual violence, history of discrimination due to gender identity in a lifetime, and weather the participant changed their name in official documents (name change) (yes, no). Descriptive statistics were employed. Bivariate and multiple logistic regression models with random intercepts to accommodate the effect of the city where the data was collected were built to investigate the association of the study variables with the prevalence of active syphilis. The model selection was performed following the recommendation for a logistic regression model^12^. Variables presenting a p-value of 0.3 or less were selected as candidates to be included in the final multiple model. The modeling process started with the full model, with all candidates, and variables were dropped one by one, aiming to minimize the Akaike Information Criterion (AIC). For this analysis, sampling weights were not used^13^. Odds ratios (OR) and 95% confidence intervals (95%CI) were estimated. All analyses were performed in R, 4.3.1^14^.

The project was approved by the Research Ethics Committee of the Santa Casa de Misericórdia de São Paulo (CAAE 05585518.7.0000.5479; opinion n°: 3.126.815 – 30/01/2019), as well as by other participating institutions. Participants provided written consent, and referrals for needed clinical and social services were made by the counselors.

## RESULTS

A total of 1,317 TGW were recruited and answered the questionnaire. Of them, 26 were excluded for not performing the diagnostic tests for syphilis. Out of the 1,291 participants included, 786 (60.9%) tested positive in the rapid tests and 294 (22.8%) were diagnosed as having active syphilis. In the cities where the study was conducted, the prevalence of active syphilis was 17.4% (95%CI 13–21) in São Paulo (SP), 20.3% (95%CI 14–27) in Porto Alegre (RS), 27.2% (95%CI 23–37) in Salvador (BA), 26.8% (95%CI 22–32) in Manaus (AM) and 21.5% (95%CI 16–29) in Campo Grande (MS).

Among the participants with active syphilis, 224 (76.2%) self-reported as black or mixed race, 195 (66.3%) were up to 34 years old, 189 (64.3%) completed high school education, 128 (43.5%) received less than one minimum wage. Most 227 (77.2%) did not change their name in official documents. The majority of participants referred sex work as an occupation, 94 (32.0%) reported having engaged in sex work in the past and 156 (53.1%) currently, with 69 (23.5%) as a part-time income and 87 (29.6%) as a main source of income (Table 1).

**Table 1:** Sociodemographic characteristic of transgender women and *travestis* with diagnosis of active syphilis in Brazil (Dec. 2019–Jul. 2021).

	n (%)	OR	95%CI	p-value
Skin color/Race				
White	59 (20.1)	1	- - -	- - -
Black/Mixed	224 (76.2)	1.41	1.01–1.97	0.04
Other	8 (2.7)	0.98	0.42–2.27	0.96
Age				
35 or more	99 (33.7)	1	- - -	- - -
Up to 34	195 (66.3)	0.99	0.74–1.32	0.95
Level of education				
Higher education or above	10 (3.4)	1	- - -	- - -
High school	189 (64.3)	1.44	0.71–2.92	0.31
Basic education	94 (32.0)	2.44	1.17–5.06	0.01
Income (minimum wages)				
Two or more	40 (13.6)	1	- - -	- - -
One to two	90 (30.6)	0.9	0.58–1.39	0.64
Less than one	128 (43.5)	0.94	0.61–1.42	0.75
Marital status				
Single	219 (74.5)	1	- - -	- - -
In a relationship	36 (12.2)	0.82	0.54–1.22	0.33
Married or *de facto*	39 (13.3)	0.94	0.63–1.40	0.77
Housing arrangement				
Owner	64 (21.8)	1	- - -	- - -
Leaser	108 (36.7)	1.19	0.83–1.70	0.34
With friends or family	85 (28.9)	1.15	0.77–1.69	0.48
Change name in official documents				
Yes	67 (22.8)	1	- - -	- - -
No	227 (77.2)	1.39	1.00–1.91	0.04
Sex work				
No	42 (14.3)	1	- - -	- - -
Past only	94 (32.0)	2.22	1.47–3.32	<0.01
Partial source of income (currently)	69 (23.5)	2.75	1.78–4.25	<0.01
Main source of income (currently)	87 (29.6)	3.62	2.36–5.53	<0.01

The vast majority, or 248 (84.4%) of the participants, reported suffering discrimination due to gender identity in a lifetime, 264 (89.8%) experienced situations of violence and 146 (49.7%) were victims of sexual assault (Table 2).

**Table 2:** Experience of violence of transgender women and *travestis* with diagnosis of active syphilis in Brazil (Dec. 2019–Jul. 2021).

	n (%)	OR	95%CI	p-value
Discrimination due to gender identity in a lifetime				
No	45 (15.3)	1.00	- - -	- - -
Yes	248 (84.4)	0.95	0.65–1.37	0.77
Assault				
No	161 (54.8)	1.00	- - -	- - -
Yes	129 (43.9)	0.86	0.65–1.12	0.26
Physical assault				
No	244 (83.0)	1.00	- - -	- - -
Yes	47 (16.0)	1	0.69–1.44	0.99
Sexual assault				
No	145 (49.3)	1.00	- - -	- - -
Yes	146 (49.7)	1.01	0.77–1.32	0.94
Violence in general				
No	28 (9.5)	1.00	- - -	- - -
Yes	264 (89.8)	0.85	0.53–1.35	0.49

Odds-ratio point estimates are represented by dots and 95% confidence intervals are represented by bars. Different colors were used for different variables to improve visualization, with the statistically significant variables colored in blue.

**Figure 1:**
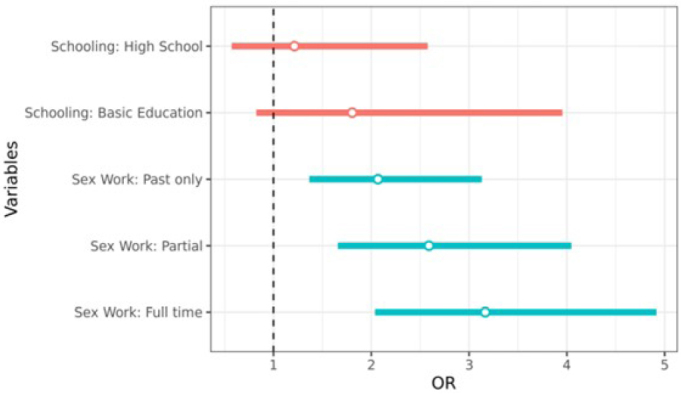
Factors associated with active syphilis in transgender women and *travestis* in a multivariate model, in Brazil (Dec. 2019–Jul. 2021).

Black/mixed race (OR=1.41, 95%CI 1.01–1.97), basic level of education (OR=2.44, 95%CI 1.17–5.06), no name change in documents (OR=1.39, 95%CI 1.00–1.91) and sex work (past only OR=2.22, 95%CI 1.47–3.32; partial OR=2.75, 95%CI 1.78–4.25; full time OR=3.62, 95%CI 2.36–5.53) were associated with active syphilis. In the multivariate analysis, sex work was associated with active syphilis with higher chances of infection for sex work in the past (OR=2.07; 95%CI 1.37–3,13), for current part-time sex work (OR=2.59; 95%CI 1.66–4.05) and for full-time sex work as the main source of income (OR=3.16; 95%CI 2.04–4.92) when compared to no sex work (Figure 1).

Odds-ratio point estimates are represented by dots and 95% confidence intervals are represented by bars. Different colors were used for different variables to improve visualization, with the statistically significant variables colored in blue.

## DISCUSSION

One in five TGW in our study were diagnosed with active syphilis, and more than half have a history of exposure to the disease, revealing that syphilis remains as an important STI diagnosis to be pursued in this population, considering the impacts of the disease progression and of lack of adequate treatment on quality of life^8^. Our estimates of seroprevalence are higher than those found in other countries of Latin America and the Caribbean, considering the seroprevalence of syphilis of 54.8% among TGW found in Peru and 47.5% in the Dominican Republic^17^, reinforcing the need to focus on diagnostic and prevention strategies in the context of Latin America.

Analyzing the sociodemographic features, the sample is composed mostly of black/mixed race TGW, around 30 years old, with basic or high-school education — characteristics similar to findings of other studies about the prevalence of HIV and STIs on this population^19^.

Stigma also can affect the cascade of sexually transmitted diseases in other aspects. As violence and discrimination keep TGW away from health services, these conditions also keep them from having adequate access to education and other basic rights, deepening their situation of social vulnerability^19,20^. The level of education is associated with the diagnosis of ‘active syphilis’, with those with lower level of education presenting a higher risk of having the disease. Although we cannot assume causality, the association highlights the importance of targeting those with lower level of education in prevention and health promotion strategies aimed at syphilis and other STIs.

In our study, sex work was associated with the diagnosis of syphilis and can be interpreted as a risk factor for active infection. As demonstrated in other studies, sex work is associated with STIs as a result of a series of unfortunate events such as unemployment, economic instability, food insecurity and stigma, leading to prostitution as a way to earn a living^23^. In this way, this population is often trapped in circles of violence and exclusion that intensifies their risk of acquiring HIV and other STIs. Furthermore, the transmission of syphilis, whose dynamics encompass not only penetration (anal or vaginal), but also oral sex and intimate contact, can be facilitated in the context of sex work, putting these professionals at greater risk of acquiring this infection.

Syphilis remains an important public health problem to be addressed, taking into consideration the specific needs of the most vulnerable groups, such as the ones approached in this study. Structural barriers need to be taken into consideration in settings like Brazil, where TGW are often marginalized and are the victims of stigma and discrimination. Actions aimed at specific populations, such as sex workers, can be interesting alternatives for policies.

Our study has limitations. This article use data from a cross-sectional study, and although risk factors associated with the diagnosis of active syphilis were identified, it was not possible to establish the directionality of such associations. The study used a non-representative sample, considering the RDS sampling method for hard-to-reach populations and using data collected in five major cities in the country. Brazil is a big, heterogeneous country, and the population may differ in some aspects among the major cities, aspects that it was not possible to address in this study. The information about sociodemographic characteristics, behaviors, access to health care and previous STIs were self-reported, favoring information bias.

To date, this is the first large study performed among transgender women and *travestis* in the five regions of Brazil, using face-to-face interviews and performing serological tests, providing data on the panorama of syphilis and presenting an estimation of other STIs in Brazil.
