# Erratum

**DOI:** 10.1590/1980-549720240003.supl.1erratum

**Published:** 2024-12-09

In the manuscript "*Prevalence of syphilis in transgender women and travestis in Brazil: results from a national cross-sectional study*", DOI: https://doi.org/10.1590/1980-549720240003.supl.1, published in the Rev Bras Epidemiol. 2024; 27(Suppl 1): e240003.supl.1.

**Where it reads:**

Maria Inês Costa Dourado

**It should read:**

Inês Dourado

