# Supplementary material for: Prevalence of syphilis in transgender women and travestis in Brazil: results from a national cross-sectional study
Source: Rev Bras Epidemiol. 2024 Aug 19;27(Suppl 1):e240003.supl.1. doi: 10.1590/1980-549720240003.supl.1 (PMC11338538; doi:10.1590/1980-549720240003.supl.1)
Supplement: Translation

# Translation: Prevalência de sífilis em mulheres trans e travestis no Brasil: resultados de um estudo multicêntrico nacional

**DOI:** 10.1590/1980-549720240003.supl.1.2

**Authors:** Aline Borges Moreira da Rocha, Sandro Sperandei, Adele Benzaken, Rita Bacuri, Katia Cristina Bassichetto, Elaine Lopes de Oliveira, Edilene Peres Real da Silveira, Maria Inês Costa Dourado, Maria Amélia de Sousa Mascena Veras

**Affiliations:** I Santa Casa de São Paulo, Faculdade de Ciências Médicas – São Paulo (SP), Brasil.; II Western Sydney University, Translational Health Research Institute – Sydney (NSW), Austrália.; III Fiocruz Amazônia, Instituto Leônidas e Maria Deane – Manaus (AM), Brasil.; IV Aids Healthcare Foundation – Los Angeles (CA), EUA.; V Instituto Adolfo Lutz – São Paulo (SP), Brasil.; VI Universidade Federal da Bahia, Instituto de Saúde Coletiva – Salvador (BA), Brasil.

**Keywords:** Sífilis, Mulheres trans, Prevenção, ISTs

## Abstract

**Objetivo:**

O estudo teve como objetivo estimar a prevalência de sífilis adquirida e fatores associados em uma pesquisa nacional.

**Métodos:**

"*TransOdara*" foi um estudo transversal compreendendo mulheres trans e travestis (MTT) em cinco grandes cidades do Brasil durante dezembro–2019 e julho–2021. A amostra foi recrutada usando o método *respondente-driven sampling* (RDS). O desfecho “sífilis ativa” foi definido como um teste treponêmico positivo e título do *Venereal-Disease-Research-Laboratory* (VDRL) maior ou igual a ⅛. Variáveis sociodemográficas foram descritas. Análises bi- e multivariadas foram realizadas, e *odds ratio* (OR) e IC95% foram estimados. Todas as análises foram realizadas no R,4.3.1.

**Resultados:**

Um total de 1.317 MTT foram recrutadas, com 1.291 sendo testadas para sífilis, das quais 294 (22,8%) preencheram os critérios para sífilis ativa. Na análise bivariada, raça negra/parda (OR=1,41; IC95% 1,01–1,97), nível básico de educação (OR=2,44; IC95% 1,17–5,06), não alteração do nome nos documentos (OR=1,39; IC95% 1,00–1,91) e trabalho sexual (pregresso OR=2,22; IC95% 1,47–3,32; parcial OR=2,75; IC95% 1,78–4,25; período integral OR=3,62; IC95%: 2,36-5,53) foram associados à sífilis ativa. Na análise multivariada, o trabalho sexual foi o único fator associado, 2,07 (IC95%: 1,37-3,13) trabalho sexual passado, 2,59 (IC95% 1,66–4,05) trabalho sexual em tempo parcial e 3,16 (IC95% 2,04–4,92) trabalho sexual como principal fonte de renda.

**Conclusão:**

A prevalência de sífilis ativa neste estudo foi elevada em comparação com outros países da América Latina. O trabalho sexual foi um fator associado importante com sífilis ativa, destacando o impacto que essa condição de vulnerabilidade pode ter na saúde das MTT, como membros de uma população-chave marginalizada.

## INTRODUÇÃO

As infecções sexualmente transmissíveis (ISTs) representam uma condição de saúde comumente identificada e afetam diferentes populações em todo o mundo. A sífilis constitui um importante problema de saúde pública a ser abordado, considerando relatos de aumento de taxas de infecção, o acesso limitado a testes rápidos (*point-of-care tests* – PoCT) em alguns países e múltiplas fases de manifestação clínica^1^. No Brasil, dados recentes do sistema nacional de vigilância relataram 1.115.529 casos de sífilis adquirida entre os anos de 2011 e 2021, com notificações concentradas em jovens do sexo masculino e taxas de detecção em constante aumento: 9,3 casos por 100 mil habitantes em 2011 e 78,5 casos por 100 mil habitantes em 2021^2^. Vale ressaltar que o sistema de vigilância do Brasil não coleta dados a respeito da identidade de gênero das pessoas infectadas.

Mulheres trans e travestis (MTT) são consideradas população-chave para infecção por HIV e outras ISTs incluindo sífilis, uma condição clínica que frequentemente não é diagnosticada, principalmente devido à dificuldade de acesso aos serviços de saúde^3^. Na América Latina, os dados sobre sífilis entre a população trans são escassos. Uma pesquisa realizada na República Dominicana encontrou uma prevalência de 47,4% para casos de sífilis em MTT, e um estudo retrospectivo de revisão de prontuários, executado no Peru, encontrou prevalência de 54,8% em Lima^4,5^. Alguns estudos brasileiros abordam a prevalência de sífilis entre a população de MTT, com dados variando de 33,3% em um estudo de coorte de PrEP entre adolescentes realizado em três capitais brasileiras entre 2019 e 2021, a 50,0% em um estudo transversal realizado com MTT na região central do Brasil em 2014^6,7^.

Entre os fatores associados a uma maior prevalência de sífilis entre as MTT, o baixo nível socioeconômico e o trabalho sexual emergem como os mais importantes. Essas condições estão intimamente relacionadas à situação de vulnerabilidade social a que uma parte substancial dessa população está sujeita, dificultando o acesso aos serviços de saúde, especialmente aqueles relacionados à saúde sexual^3,8^.

O presente estudo estimou a prevalência de sífilis adquirida, sífilis ativa e fatores associados entre MTT no Brasil.

## MÉTODOS

O TransOdara foi um estudo transversal envolvendo MTT conduzido em cinco grandes cidades em todas as regiões do Brasil: Campo Grande (MS), Manaus (AM), Porto Alegre (RS), Salvador (BA) e São Paulo (SP), entre novembro de 2019 e julho de 2021.

As MTT foram recrutadas usando o método de amostragem *Respondent-driven sample* (RDS), uma abordagem usada para atingir populações de difícil acesso que depende de redes sociais para recrutar membros da uma mesma população^9^. Para este estudo, o recrutamento ocorreu entre dezembro de 2019 e julho de 2021, considerando as possíveis variações em cada local devido ao enorme impacto da pandemia de COVID-19 em todos os aspectos dos serviços de saúde.

Todas as participantes responderam a um questionário estruturado sobre aspectos sociodemográficos, experiências de estigma e discriminação e conhecimentos prévios sobre HIV e outras ISTs, incluindo informações sobre testes e tratamentos anteriores de cada uma delas. Foram oferecidos testes rápidos de sangue para HIV, sífilis e hepatites A, B e C, além de exame PCR em tempo real (*Abbott Real Time CT/NG Controls*) para *Neisseria gonorrhoeae* e *Chlamydia trachomatis*, nas amostras de *swabs* anais e genitais/uretrais autocoletadas, com testes confirmatórios em caso de resultado positivo e oferecimento de tratamento para casos confirmados. No caso de participantes sintomáticas para diferentes ISTs, após a coleta de amostras, foi realizada uma abordagem sindrômica, com base nas diretrizes do Ministério da Saúde, e prescrito o tratamento adequado. Para mais detalhes metodológicos do estudo, ver o artigo metodológico sobre o TransOdara publicado neste mesmo suplemento^10^.

Variáveis do estudo: Para o desfecho “sífilis ativa”, amostras de sangue foram testadas no Instituto Adolfo Lutz, um laboratório público de referência em saúde pública do estado de São Paulo. Para o diagnóstico da sífilis, foram utilizados um teste rápido (treponêmico) fornecido pelo programa de saúde estadual e um teste não treponêmico, o *Venereal Disease Research Laboratory* (VDRL) para confirmação. Em caso de resultados inconsistentes (teste rápido negativo e VDRL positivo), um segundo teste treponêmico (*fluorescent treponemal antibody absorption test* – FTA-Abs) foi realizado para confirmar os resultados. As participantes que apresentaram teste treponêmico positivo e títulos de VDRL maiores ou iguais a ⅛ foram classificadas como portadoras de sífilis ativa para esta análise e receberam tratamento. Devido à dificuldade de caracterização do acompanhamento clínico das participantes com títulos de VDRL inferiores a ⅛, essas mulheres também receberam tratamento, de acordo com o manual do Ministério da Saúde para tratamento de ISTs^11^.

As variáveis incluídas nas análises de fatores associados foram: idade (até 34 anos *vs*. 35 ou mais), estado civil (solteira, incluindo separada, divorciada ou viúva, em um relacionamento estável, mas não casada, e casada), raça/cor da pele (branca, negra ou parda e outras), maior nível de escolaridade (básico, médio e superior), situação habitacional (proprietária, aluguel, com amigos ou familiares e outros, incluindo moradoras de rua, hotéis e instituições), renda mensal (menos de um salário mínimo, um a dois salários mínimos e mais de dois salários mínimos — considerando o salário mínimo equivalente a 218,29 dólares americanos), histórico de trabalho sexual (nunca, apenas no passado, atualmente como fonte de renda parcial e atualmente como fonte de renda principal), histórico de violência, histórico de agressão física, histórico de violência sexual, histórico de discriminação devido à identidade de gênero na vida e se a participante alterou seu nome em documentos oficiais (mudança de nome) (sim, não) . Estatísticas descritivas foram empregadas. Modelos de regressão logística bivariada e múltipla com interceptos aleatórios para acomodar o efeito do município onde os dados foram coletados foram construídos para investigar a associação das variáveis do estudo com a prevalência de sífilis ativa. A seleção do modelo foi realizada seguindo recomendação de modelo de regressão logística^12^. Variáveis com valor de p menor ou igual a 0,3 foram selecionadas como candidatas a serem incluídas no modelo múltiplo final. O processo de modelagem iniciou-se com o modelo completo, com todas as candidatas, e as variáveis foram eliminadas uma a uma, com o objetivo de minimizar o Critério de Informação de Akaike (*Akaike Information Criterion* – AIC). Para esta análise, não foram utilizados pesos amostrais^13^. Foram estimados *odds ratio* (OR) e intervalos de confiança de 95% (IC 95%). Todas as análises foram realizadas em R, 4.3.1^14^.

O projeto foi aprovado pelo Comitê de Ética em Pesquisa (CEP) da Santa Casa de Misericórdia de São Paulo (CAAE 05585518.7.0000.5479 - Nº parecer: 3.126.815 - 30/01/2019), assim como pelas demais instituições participantes. As participantes forneceram consentimento por escrito e os encaminhamentos para os serviços clínicos e sociais necessários foram feitos pelos orientadores.

## RESULTADOS

Um total de 1.317 MTT foram recrutadas e responderam ao questionário. Dessas, 26 foram excluídas por não realizarem os testes diagnósticos para sífilis. Das 1.291 participantes incluídas, 786 (60,9%) testaram positivo nos testes rápidos e 294 (22,8%) foram diagnosticadas com sífilis ativa. Nas cidades onde o estudo foi realizado, a prevalência de sífilis ativa foi de 17,4% (IC 95% 13–21) em São Paulo (SP), 20,3% (IC 95% 14–27) em Porto Alegre (RS), 27,2 % (IC 95% 23–37) em Salvador (BA), 26,8% (IC 95% 22–32) em Manaus (AM) e 21,5% (IC 95% 16–29) em Campo Grande (MS).

Entre as participantes com sífilis ativa, 224 (76,2%) se autodeclararam pretas ou pardas, 195 (66,3%) tinham até 34 anos, 189 (64,3%) completaram o ensino médio, 128 (43,5%) recebiam menos de um salário mínimo. A maioria, 227 (77,2%), não alterou o nome em documentos oficiais. A maioria das participantes referiu o trabalho sexual como uma ocupação, 94 (32,0%) relataram ter praticado trabalho sexual no passado e 156 (53,1%) atualmente, sendo 69 (23,5%) como uma fonte de renda parcial e 87 (29,6%) como fonte de renda principal (Tabela 1).

**Tabela 1:** Característica sociodemográfica de mulheres trans e travestis com diagnóstico de sífilis ativa no Brasil (dez. 2019–jul. 2021).

	n (%)	OR	IC 95%	Valor de p
Cor da pele/Raça				
Branca	59 (20,1)	1	- - -	- - -
Negra/Parda	224 (76,2)	1,41	1,01–1,97	0,04
Outra	8 (2,7)	0,98	0,42–2,27	0,96
Idade				
35 ou mais	99 (33,7)	1	- - -	- - -
Até 34 anos	195 (66,3)	0,99	0,74–1,32	0,95
Nível de escolaridade				
Ensino superior ou mais	10 (3,4)	1	- - -	- - -
Ensino médio	189 (64,3)	1,44	0,71–2,92	0,31
Ensino fundamental	94 (32,0)	2,44	1,17–5,06	0,01
Renda (salário mínimo)				
Dois ou mais	40 (13,6)	1	- - -	- - -
Um a dois	90 (30,6)	0,9	0,58–1,39	0,64
Menos que um	128 (43,5)	0,94	0,61–1,42	0,75
Estado civil				
Solteira	219 (74,5)	1	- - -	- - -
Em um relacionamento	36 (12,2)	0,82	0,54–1,22	0,33
Casada ou *de facto*	39 (13,3)	0,94	0,63–1,40	0,77
Situação de moradia				
Própria	64 (21,8)	1	- - -	- - -
Aluguel	108 (36,7)	1,19	0,83–1,70	0,34
Com amigos ou família	85 (28,9)	1,15	0,77–1,69	0,48
Mudança de nome em documentos oficiais				
Sim	67 (22,8)	1	- - -	- - -
Não	227 (77,2)	1,39	1,00–1,91	0,04
Trabalho sexual				
Não	42 (14,3)	1	- - -	- - -
Apenas no passado	94 (32,0)	2,22	1,47–3,32	<0,01
Fonte de renda parcial (atualmente)	69 (23,5)	2,75	1,78–4,25	<0,01
Fonte de renda principal (atualmente)	87 (29,6)	3,62	2,36–5,53	<0,01

A grande maioria, 248 (84,4%) das participantes, relatou ter sofrido discriminação devido à identidade de gênero ao longo da vida, 264 (89,8%) vivenciaram situações de violência e 146 (49,7%) foram vítimas de agressão sexual (Tabela 2).

**Tabela 2:** Experiência de violência de mulheres trans e travestis com diagnóstico de sífilis ativa no Brasil (dez. 2019–jul. 2021).

	n (%)	OR	IC 95%	Valor de p
Discriminação devido à identidade de gênero ao longo da vida				
Não	45 (15,3)	1,00	- - -	- - -
Sim	248 (84,4)	0,95	0,65–1,37	0,77
Agressão				
Não	161 (54,8)	1,00	- - -	- - -
Sim	129 (43,9)	0,86	0,65–1,12	0,26
Agressão física				
Não	244 (83,0)	1,00	- - -	- - -
Sim	47 (16,0)	1	0,69–1,44	0,99
Agressão sexual				
Não	145 (49,3)	1,00	- - -	- - -
Sim	146 (49,7)	1,01	0,77–1,32	0,94
Violência em geral				
Não	28 (9,5)	1,00	- - -	- - -
Sim	264 (89,8)	0,85	0,53–1,35	0,49

Estimativas pontuais de *odds ratio* s⭠representadas por pontos e intervalos de confian柠de 95% s⭠representados por barras. Cores diferentes foram usadas para vari൥is diferentes para melhorar a visualiza袯, com as vari൥is estatisticamente significativas coloridas em azul.

Raça preta/parda (OR=1,41; IC 95% 1,01–1,97), escolaridade básica (OR=2,44; IC 95% 1,17–5,06), ausência de alteração de nome em documentos (OR=1,39; IC 95% 1,00–1,91) e trabalho sexual (somente no passado OR=2,22, IC 95% 1,47–3,32; parcial OR=2,75, IC 95% 1,78–4,25; tempo integral OR=3,62, IC 95% 2,36–5,53) estiveram associados à sífilis ativa. Na análise multivariada, o trabalho sexual foi associado à sífilis ativa com maiores chances de infecção para trabalho sexual no passado (OR=2,07; IC 95% 1,37–3,13), para trabalho sexual atual em período parcial (OR=2,59; IC 95% 1,66–4,05) e para trabalho sexual em período integral como principal fonte de renda (OR=3,16; IC 95% 2,04–4,92) em comparação com a ausência de trabalho sexual (Figura 1).

**Figura 1:** Fatores associados à sífilis ativa em mulheres trans e travestis em um modelo multivariado, no Brasil (dez. 2019–jul. 2021).

Estimativas pontuais de odds ratio são representadas por pontos e intervalos de confiança de 95% são representados por barras. Cores diferentes foram usadas para variáveis diferentes para melhorar a visualização, com as variáveis estatisticamente significativas coloridas em azul.

## DISCUSSÃO

Uma em cada cinco MTT em nosso estudo foi diagnosticada com sífilis ativa, e mais da metade tem histórico de exposição à doença, revelando que a sífilis permanece como um importante diagnóstico de IST para essa população, considerando os impactos da progressão da doença e da falta de tratamento adequado na qualidade de vida^8^. Nossas estimativas de soroprevalência são superiores às encontradas em outros países da América Latina e do Caribe, considerando a soroprevalência de sífilis de 54,8% entre MTT observada no Peru e 47,5% na República Dominicana^17^, reforçando a necessidade de foco em estratégias de diagnóstico e prevenção no contexto da América Latina.

Analisando as características sociodemográficas, a amostra é composta principalmente por MTT pretas/pardas, com idade em torno de 30 anos, com ensino fundamental ou médio — características semelhantes às encontradas em outros estudos sobre prevalência de HIV e ISTs nessa população^19^.

O estigma também pode afetar a cascata de doenças sexualmente transmissíveis em outros aspectos. À medida que a violência e a discriminação afastam as MTT dos serviços de saúde, essas condições também as impedem de ter acesso adequado à educação e a outros direitos básicos, aprofundando a sua situação de vulnerabilidade social^19,20^. O nível de escolaridade está associado ao diagnóstico de “sífilis ativa”, sendo que aqueles com menor escolaridade apresentam maior risco de ter a doença. Embora não possamos assumir causalidade, a associação destaca a importância de direcionar aquelas com menor nível de escolaridade nas estratégias de prevenção e promoção da saúde voltadas à sífilis e outras ISTs.

Em nosso estudo, o trabalho sexual esteve associado ao diagnóstico de sífilis e pode ser interpretado como um fator de risco para infecção ativa. Como demonstrado em outros estudos, o trabalho sexual está associado a ISTs como resultado de uma série de acontecimentos adversos, como desemprego, instabilidade econômica, insegurança alimentar e estigma, levando à prostituição como forma de ganhar a vida^23^. Dessa forma, essa população fica muitas vezes presa em círculos de violência e exclusão que intensificam o risco de adquirir HIV e outras ISTs. Além disso, a transmissão da sífilis, cuja dinâmica abrange não apenas a penetração (anal ou vaginal), mas também o sexo oral e o contato íntimo, pode ser facilitada no contexto do trabalho sexual, colocando essas profissionais em maior risco de adquirir infecção.

A sífilis continua sendo um importante problema de saúde pública a ser enfrentado, levando-se em consideração as necessidades específicas dos grupos mais vulneráveis, como os abordados neste estudo. As barreiras estruturais precisam ser levadas em consideração em contextos como o do Brasil, onde as MTT são frequentemente marginalizadas e vítimas de estigma e discriminação. Ações voltadas para populações específicas, como as profissionais do sexo, podem ser alternativas interessantes de políticas de saúde pública.

Nosso estudo apresenta limitações. Este artigo utilizou dados provenientes de estudo transversal e, embora tenham sido identificados fatores de risco associados ao diagnóstico de sífilis ativa, não foi possível estabelecer efeito causal para tais associações. O estudo utilizou uma amostra não representativa, considerando o método de amostragem RDS para populações de difícil acesso, utilizando também dados coletados em cinco grandes cidades do país. O Brasil é um país grande e heterogêneo, e a população pode diferir em alguns aspectos entre as principais cidades, aspectos que não foram possíveis de abordar neste estudo. As informações sobre características sociodemográficas, comportamentos, acesso aos cuidados de saúde e ISTs anteriores foram autorreferidas, favorecendo o viés de informação.

De toda forma, até o momento, este é o primeiro estudo de grande porte realizado entre mulheres trans e travestis nas cinco regiões do Brasil, utilizando entrevistas presenciais e realizando testes sorológicos, fornecendo dados sobre o panorama da sífilis e apresentando uma estimativa de outras ISTs no Brasil.
