# Supplementary material for: Erratum
Source: Rev Bras Epidemiol. 2024 Dec 9;27:e2023470erratum. doi: 10.1590/1980-549720240003.supl.1erratum (PMC11656494; doi:10.1590/1980-549720240003.supl.1erratum)
Supplement: Related texts

# Correction: Errata

**DOI:** 10.1590/1980-549720240003.supl.1.2erratum

No artigo "*Prevalência de sífilis em mulheres trans e travestis no Brasil: resultados de um estudo multicêntrico nacional*", DOI: https://doi.org/10.1590/1980-549720240003.supl.1.2, publicado no periódico Rev Bras Epidemiol. 2024; 27(Suppl 1): e240003.supl.1

**Onde se lia:**

Maria Inês Costa Dourado

**Leia-se:**

Inês Dourado
